# Patient Enrolment into HIV Care and Treatment within 90 Days of HIV Diagnosis in Eight Rwandan Health Facilities: A Review of Facility-Based Registers

**DOI:** 10.1371/journal.pone.0036792

**Published:** 2012-05-11

**Authors:** Felix R. Kayigamba, Mirjam I. Bakker, Hadassa Fikse, Veronicah Mugisha, Anita Asiimwe, Maarten F. Schim van der Loeff

**Affiliations:** 1 INTERACT, Kigali, Rwanda; 2 Royal Tropical Institute, KIT Biomedical Research, Amsterdam, The Netherlands; 3 ICAP, Mailman School of Public Health, Columbia University, Kigali, Rwanda; 4 Rwanda Biomedical Centre (RBC), Institute of HIV Disease Prevention and Control, Ministry of Health, Kigali, Rwanda; 5 Amsterdam Institute of Global Health and Development (AIGHD), Amsterdam, The Netherlands; 6 Centre for Infection and Immunity Amsterdam (CINIMA), AMC, Amsterdam, The Netherlands; 7 Public Health Service of Amsterdam (GGD), Amsterdam, The Netherlands; University of California, San Francisco, United States of America

## Abstract

**Introduction:**

Access to antiretroviral therapy (ART) has increased greatly in sub-Saharan Africa. However many patients do not enrol timely into HIV care and treatment after HIV diagnosis. We studied enrolment into care and treatment and determinants of non-enrolment in Rwanda.

**Methods:**

Data were obtained from routine clinic registers from eight health facilities in Rwanda on patients who were diagnosed with HIV at the antenatal care, voluntary counselling-and-testing, outpatient or tuberculosis departments between March and May 2009. The proportion of patients enrolled into HIV care and treatment was calculated as the number of HIV infected patients registered in ART clinics for follow-up care and treatment within 90 days of HIV diagnosis divided by the total number of persons diagnosed with HIV in the study period.

**Results:**

Out of 482 patients diagnosed with HIV in the study period, 339 (70%) were females, and the median age was 29 years (interquartile range [IQR] 24–37). 201 (42%) enrolled into care and treatment within 90 days of HIV diagnosis. The median time between testing and enrolment was six days (IQR 2–14). Enrolment in care and treatment was not significantly associated with age, sex, or department of testing, but was associated with study site. None of those enrolled were in WHO stage 4. The median CD4 cell count among adult patients was 387 cells/mm^3^ (IQR: 242–533 cells/mm^3^); 81 of 170 adult patients (48%) were eligible to start ART (CD4 count<350 cells/mm^3^ or WHO stage 4). Among those eligible, 45 (56%) started treatment within 90 days of HIV diagnosis.

**Conclusion:**

Less than 50% of diagnosed HIV patients from eight Rwandan health facilities had enrolled into care and treatment within 90 days of diagnosis. Improving linkage to care and treatment after HIV diagnosis is needed to harness the full potential of ART.

## Introduction

In many sub Saharan African countries access to antiretroviral therapy (ART) has increased greatly in recent years [1], [2]. Linkage to care and treatment after a positive HIV test is crucial to harness the potential of ART; studies have shown that mortality among diagnosed HIV patients awaiting ART can be unacceptably high [3]–[5]. Enrolment into HIV care has been studied in several Sub African settings and the proportion of HIV positive patients enrolled into HIV care varied from a low 38% in a study from urban Zambia [6] to 68% six months after HIV diagnosis in rural northern Tanzania [7] and south-west Uganda [8].

Rwanda adopted a national ART program in 2003 and since then access to ART has increased fast. In Rwanda ART is available from 295 clinics, both urban and rural, throughout the country [9]. Rwanda is a densely populated country and the average distance to clinics is short compared to most sub Saharan countries. It was estimated that by the end of 2010, about 80% of HIV infected eligible patients were on ART [2], [9].

In order to ensure a timely diagnosis of HIV for all, the Ministry of Health (MOH) in Rwanda has planned the introduction of provider initiated testing (PIT) in all its health facilities. Prior to this, we studied linkage to care in four clinics in the capital Kigali, and four in the northern district Musanze, to serve as baseline measurement prior to introduction of PIT. We examined the proportion of HIV infected patients that enrolled at ART clinics, the proportion of enrolled patients that were eligible for ART, and the proportion of eligible patients that started ART, all within 90 days of diagnosis, and we examined clinic and patient factors associated with enrolment, eligibility and start of ART.

**Figure 1:**
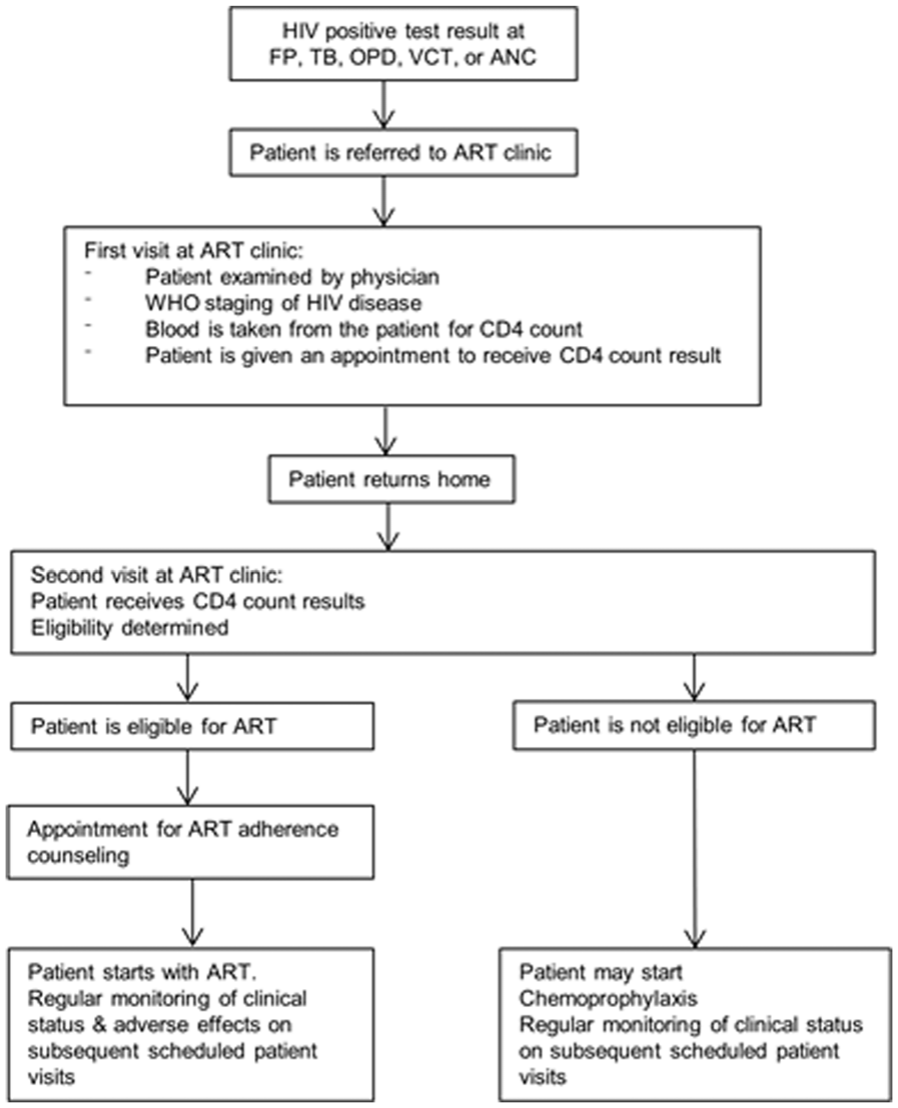
Routine flow of patients after HIV diagnosis, Rwanda, 2009.

**Figure 2:**
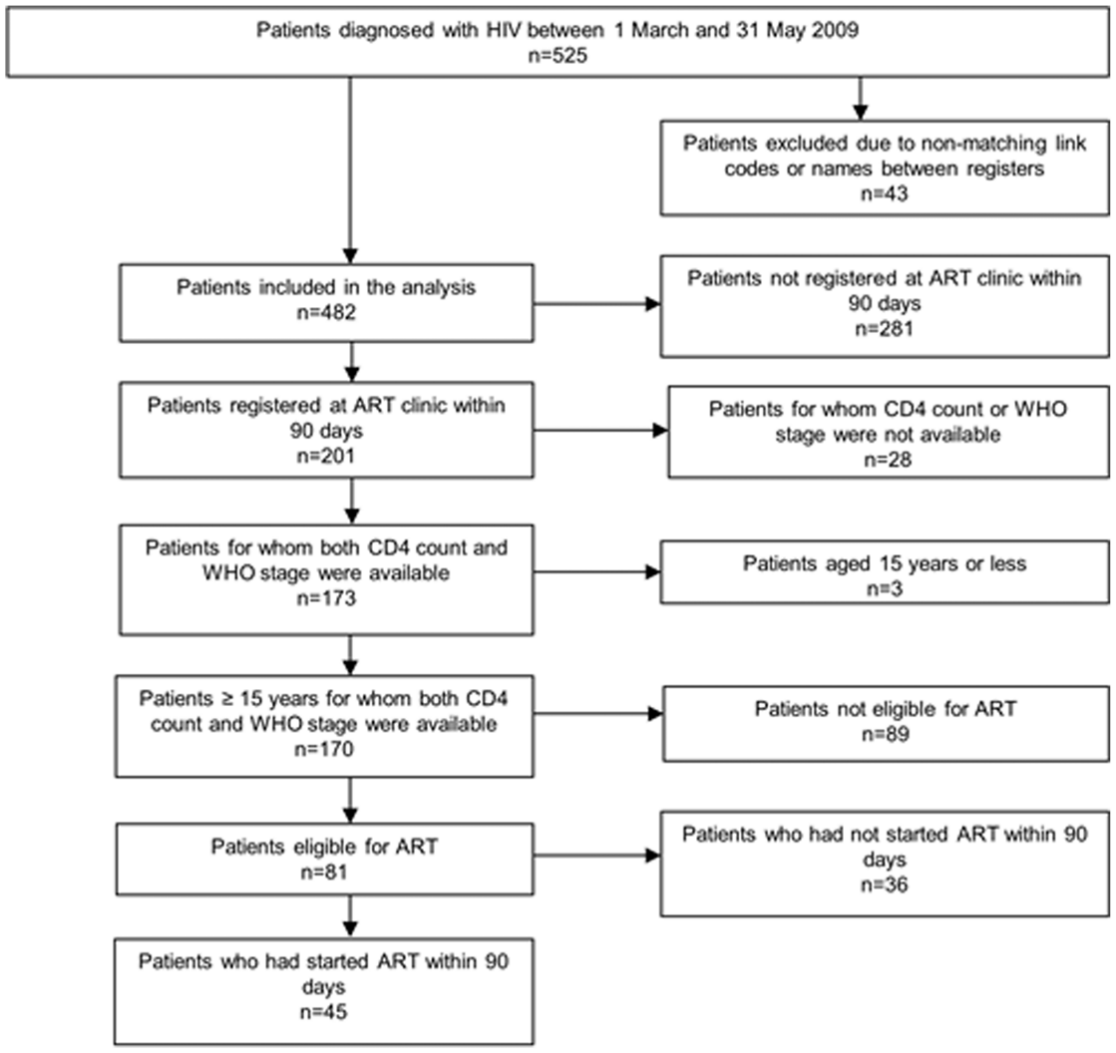
Flow of HIV positive patients' enrolment into HIV care and treatment, Rwanda, 2009.

## Methods

### Study setting

Data were obtained from facility-based registers in eight health facilities in Rwanda: four facilities in Musanze district in the North-West (Ruhengeri hospital, Muhoza health centre (HC), Gasiza HC and Rwaza HC) and four facilities from Gasabo district in the capital Kigali (Kibagabaga hospital, Kimironko HC, Kinyinya HC and Kabuye HC). Data were studied from adults and children aged 18 months or above registered at the out-patient department (OPD), voluntary counselling and testing (VCT) clinic, tuberculosis (TB) clinic and the antenatal care (ANC) clinic of the eight health facilities who were diagnosed with HIV between 1^st^ March and 31^st^ May 2009.

**Table 1:** Number of newly diagnosed HIV infected patients, by department, sex, age and study site, Rwanda, March-May 2009.

	Study sites								Total (%)
	Muhoza	Ruhengeri	Gasiza	Kibagabaga	Kinyinya	Kimironko	Rwaza	Kabuye	
Total	154	6	17	85	50	99	24	47	482 (100)
**Department**									
VCT	117	NA	10	34	26	62	5	39	293 (60.8)
ANC	31	NA	2	11	17	30	0	8	99 (20.5)
TB	NA	3	0	3	3	0	1	0	10 (2.1)
OPD	6	3	5	37	4	7	18	0	80 (16.6)
**Age group**									
0–4 years	3	0	0	1	1	1	1	0	7 (1.5)
5–14 years	1	0	0	0	1	0	0	1	3 (0.6)
15–24 years	47	0	1	20	12	31	2	8	121 (25.6)
25–34 years	65	1	6	34	20	40	4	22	192 (40.6)
35–44 years	26	2	7	8	12	19	10	13	97 (20.5)
>45 years	12	3	3	13	4	8	7	3	53 (11.2)
Missing	0	0	0	9	0	0	0	0	9
**Sex**									
Male	32	2	4	34	17	23	14	17	143 (29.7)
Female	122	4	13	51	33	76	10	30	339 (70.3)

NA = Not Applicable; TB Tuberculosis; ANC Antenatal care; HIV Human immunodefiency virus; OPD Outpatient department; VCT Voluntary counselling and testing.

**Figure 3:**
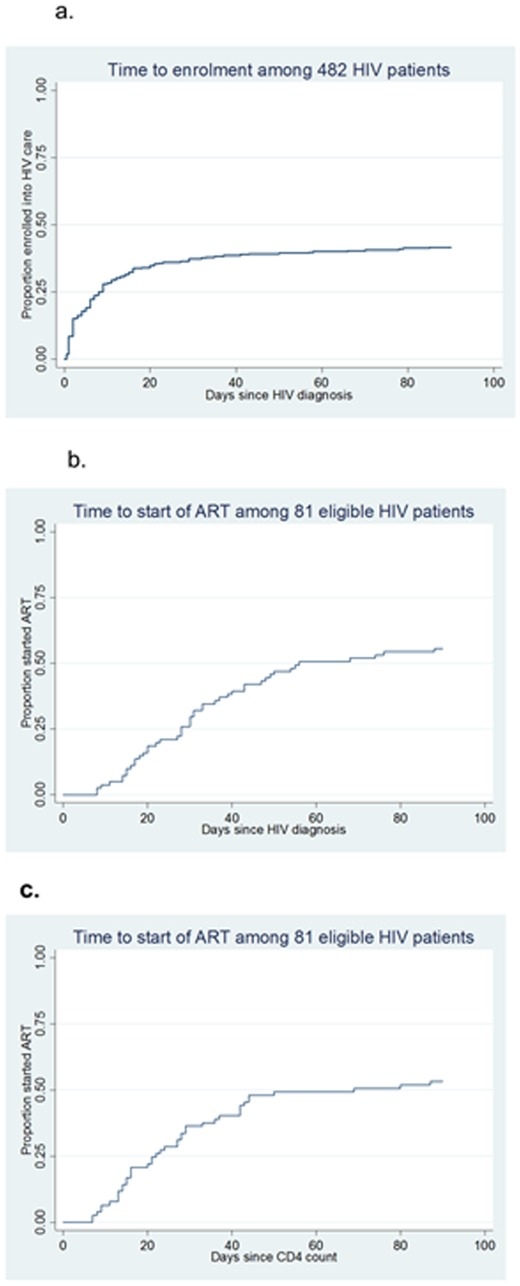
**a**. Time to enrolment among 482 HIV patients. **b**. Time to start of ART among 81 eligible HIV patients, from date of HIV diagnosis. **c**. Time to start of ART among 81 eligible HIV patients, from date of CD4 test.

### Routine practice for HIV diagnosis and ART eligibility

In each of the eight health facilities HIV counselling and testing was available. In most clinics the health worker referred the patient to the laboratory, where a blood sample was taken and an HIV test done, making use of an algorithm of three rapid tests [10]. A patient who was diagnosed with HIV was referred to the ART clinic within the same health facility, with the exception of one clinic (Muhoza), from where patients were referred to the neighbouring Ruhengeri hospital. After registration the patient was examined by a physician who established in which WHO stage the patient was [11]. A blood sample was taken to measure the CD4 count. The patient was given an appointment to receive the CD4 count result and advice regarding follow-up care and treatment (Figure 1). At the time of the study, an HIV infected patient (adult or child aged above 5 years) was eligible for ART when the CD4 count was below 350 cells/mm^3^ or was in WHO clinical stage 4 [11]. The criteria for children below 5 years of age were: CD4 count below 1500 cells/mm^3^ (children below 11 months), CD4 count below 750 cells/mm^3^ (children aged 12–35 months) and CD4 count below 350 cells/mm^3^ (children aged 36–59 months), and/or WHO clinical stage 3 or 4 [11]. Pulmonary TB was regarded as a stage 3 disease. For TB patients newly diagnosed with HIV the guideline advised to start ART 15 days after the start of TB treatment if the CD4 count was <50 cells/mm^3^, between 15 days and 2 months after the start of TB treatment if the CD4 count was between 50 and 200 cells/mm^3^, and 2 months after the start of TB treatment if the CD4 count between 200 and 350 cells/mm^3^ [12]. Before ART is started in Rwanda social support must be ensured; this means that HIV status should be disclosed to a family member or another close person. Some patients diagnosed with HIV at TB departments were being investigated for TB, but were not diagnosed with TB.

**Table 2:** Enrolment into HIV care & treatment, WHO stage, median CD4 count, ART-eligibility and start of ART by study site, department, age and sex, Rwanda, 2009.

	Total	Number (%) with 1^st^ visit to ART clinic	Number (%) with WHO stage 3 or 4a,b	Median CD4 count in cells/mm^3^ (IQR)^c^	Number (%) of ART-eligible patients^d^	Number (%) of patients started ART ≤90 days^e^
Total	482	201/482 (41.7)	28/176 (15.9)	387 (242–533)	81/170 (47.7)	45/81 (55.6)
**Study site**						
Muhoza	154	37/154 (24.0)	6/37 (16.2)	366 (247–498)	20/37 (54.1)	13/20 (65.0)
Ruhengeri	6	6/6 (100.0)	4/6 (66.7)	264 (97–440)	5/6 (83.3)	1/5 (20.0)
Gasiza	17	6/17 (35.3)	3/6 (50.0)	299 (112–417)	4/6 (66.7)	4/4 (100.0)
Kibagabaga	85	38/85 (44.7)	1/28 (3.6)	431 (258–551)	9/28 (32.1)	9/9 (100.0)
Kinyinya	50	21/50 (42.0)	2/18 (11.1)	381 (96–492)	6/14 (42.9)	4/6 (66.7)
Kimironko	99	62/99 (62.6)	8/56 (14.3)	392 (273–536)	26/54 (48.2)	5/26 (19.2)
Rwaza	24	12/24 (50.0)	3/11 (27.3)	369 (160–388)	6/11 (54.6)	4/6 (66.7)
Kabuye	47	19/47 (40.4)	1/14 (7.1)	588 (288–824)	5/14 (35.7)	5/5 (100.0)
*p-value* f		*<0.001*	*0.005*	*0.179*	*0.314*	*<0.001*
**Department**						
VCT	293	113/293 (38.6)	15/101 (14.9)	405 (279–564)	43/99 (44.3)	23/43 (53.5)
ANC	99	46/99 (46.5)	1/38 (2.6)	469 (269–565)	11/37 (29.7)	7/11 (63.6)
TB	10	7/10 (70.0)	4/7 (57.1)	188 (22–340)	6/7 (85.7)	2/6 (33.0)
OPD	80	35/80 (43.8)	8/30 (26.7)	287 (112–387)	21/29 (72.4)	13/21 (61.9)
*p-value* f		*0.139*	*0.001*	*0.0002*	*<0.001*	*0.644*
**Age**						
0–4	7	1/7 (14.3)	N.A.	N.A.	N.A.	N.A.
5–14	3	2/3 (66.7)	N.A.	N.A.	N.A.	N.A.
15–24	121	51/121 (42.2)	4/44 (9.1)	476 (349–623)	11/42 (26.2)	5/11 (45.5)
25–34	192	79/192 (41.2)	8/69 (11.6)	422 (266–583)	31/68 (45.6)	15/31 (48.4)
35–44	97	39/97 (40.2)	4/35 (11.4)	344 (146–441)	18/33 (54.6)	12/18 (66.7)
>45	53	29/53 (54.7)	12/28 (42.9)	325 (192–378)	21/27 (77.8)	13/21 (61.9)
*p-value* f		*0.253*	*0.002*	*0.0010*	*<0.001*	*0.518*
**Sex**						
Female	339	139/339 (41.0)	16/120 (13.3)	412 (259–558)	48/116 (41.4)	30/48 (62.5)
Male	143	62/143 (43.4)	12/56 (21.4)	330 (209–479)	33/54 (61.1)	15/33 (45.5)
*p-value* f		*0.632*	*0.171*	*0.0126*	*0.016*	*0.129*

N.A. Not Applicable; TB Tuberculosis; ANC Antenatal care; HIV Human immunodefiency virus; OPD Outpatient department; VCT Voluntary counselling and testing; ART antiretroviral treatment; IQR interquartile range; WHO World Health Organization.

a WHO stage was available for 179. Data provided are for the 176 patients aged 15 years or above.

b Patients with pulmonary TB and HIV infection are by definition in WHO stage 3; however not all patients diagnosed at TB clinics were confirmed TB cases.

c CD4 count results were available for 188/201. Data provided are on the 185 patients aged 15 years or above.

d ART eligibility was based on CD4 count (CD4<350 cells/mm^3^) and WHO stage (stage 4). Data provided are on the 170 patients aged 15 years or above.

e Among those who were eligible for ART. Data provided are on the 81 patients aged 15 years or above.

f The *p*-values are based on chi-squared test, Fisher's exact test or Kruskal-Wallis test, as appropriate.

### Data abstraction

For the purpose of the current study, registers from the above mentioned departments, clinics and laboratories were abstracted using structured forms. Link codes that appeared both in the laboratory and the clinical registers were used to identify HIV infected patients. ART clinics did not use the same link codes. Registers from diagnosing departments were extracted for the period 1^st^ March to 31^st^ May 2009, and a list of all patients diagnosed with HIV during the study period (including, among other demographic information, the name of the patient) was generated to enable linkage between the testing clinic and the ART clinic registers. The ART clinic registers were abstracted for the period 1^st^ March to 31^st^ August 2009 using a standardised form. After data abstraction, the names of study participants were erased from the forms.

**Table 3:** Factors associated with non-enrolment, high WHO stage and CD4 count <350 cells/mm^3^ among adult HIV patients diagnosed in eight Rwanda health facilities, 2009: logistic regression.

	Non enrolment		WHO stage 3 or 4		CD4 count <350 cells/mm^3^	
	n/N	OR (95%CI)	n/N	OR (95%CI)	n/N	OR (95%CI)
	274/472		28/176		79/185	
**Study sites**						
Muhoza	113/150	1	6/37	1	17/37	1
Ruhengeri	0/6	N.A.	4/6	10.3 (1.5–69.7)	4/6	2.4 (0.4–14.5)
Gasiza	11/17	0.6 (0.2–1.7)	3/6	5.2 (0.8–32.0)	4/6	2.4 (0.4–14.5)
Kibagabaga	46/84	0.4 (0.2–0.7)	1/28	0.2 (0.02–1.7)	13/34	0.7 (0.3–1.9)
Kinyinya	28/48	0.5 (0.2–0.9)	2/18	0.6 (0.1–3.6)	6/14	0.9 (0.3–3.1)
Kimironko	36/98	0.2 (0.1–0.3)	8/56	0.9 (0.3–.2.7)	25/60	0.8 (0.4–1.9)
Rwaza	12/23	0.4 (0.1–0.9)	3/11	1.9 (0.4–9.5)	5/11	1.0 (0.3–3.8)
Kabuye	28/46	0.5 (0.3–1.0)	1/14	0.4 (0.04–3.6)	5/17	0.5 (0.1–1.7)
*p*-value		*<0.0001*		*0.011*		*0.712*
**Departments**						
VCT	174/285	1	15/101	1	40/104	1
ANC	53/99	0.7 (0.5–1.2)	1/38	0.15 (0.02–1.2)	14/43	0.8 (0.4–1.6)
TB	3/10	0.3 (0.1–1.1)	4/7	7.6 (1.6–37.6)	6/7	9.6 (1.1–82.7)
OPD	44/78	0.8 (0.5–1.4)	8/30	2.1 (0.8–5.5)	19/31	2.5 (1.1–5.8)
*p-value*		*0.159*		*0.0013*		*0.0058*
**Age**						
15–24 years	70/121	1.7 (0.9–3.2)	4/44	0.1 (0.04–0.5)	12/46	0.2 (0.07–0.5)
25–34 years	113/192	1.7 (0.9–3.2)	8/69	0.2 (0.1–0.5)	31/76	0.4 (0.2–0.9)
35–44 years	58/97	1.8 (0.9–3.5)	4/35	0.2 (0.05–0.6)	18/35	0.6 (0.2–1.6)
>45 years	24/53	1	12/28	1	18/28	1
*p-value*		*0.316*		*0.002*		*0.0076*
**Sex**						
Female	195/333	1	16/120	1	48/128	1
Male	79/139	0.9 (0.6–1.4)	12/56	1.8 (0.8–4.1)	31/57	2.0 (1.1–3.7)
*p-value*		*0.730*		*0.180*		*0.0335*

Those aged <15 years were excluded from these analyses. NA = Not Applicable; TB Tuberculosis; ANC Antenatal care; HIV Human immunodefiency virus; OPD Outpatient department; VCT Voluntary counselling and testing; WHO World Health Organization.

Data on the following variables were abstracted: study site (health facility), department of HIV testing, age, sex, whether data on the patient were found in the ART register (yes/no), date of first visit at ART clinic, CD4 count, date of CD4 test, WHO stage, date of WHO staging, date of start of ART (if applicable). Data were entered by data entry clerks into EpiData 3.1 (EpiData Association 2008, Odense, Denmark); subsequently the electronic data were checked against the data collection forms by one of the investigators. We used Stata version 9 (Stata Corporation, College Station, TX, USA) for all data analyses.

### Analysis

The proportion of patients enrolled into HIV care and treatment was calculated as the number of HIV infected patients registered in ART clinic registers within 90 days of their HIV diagnosis divided by the total number of persons diagnosed in the study period. The proportion of patients that was eligible for ART was calculated as the number of patients who were eligible based on WHO stage or CD4 count, divided by the total number of patients of whom WHO stage and CD4 count were available. Finally, the proportion of patients who started ART within 90 days of HIV diagnosis was determined by dividing the number of eligible patients who started ART within 90 days by the number eligible for ART.

Associations between age group, sex, department of HIV diagnosis, study site and five outcomes (enrolled in care; CD4 count; WHO stage; eligible for ART; started ART) were assessed using the chi-squared test, Fisher's exact test or Kruskal-Wallis test, as appropriate.

Multivariable logistic regression analyses were done to identify factors associated with patient enrolment in care and treatment, advanced clinical disease stage (WHO stage 3 or 4) and low CD4 count (<350 cells/mm^3^). Because we assessed the effect of only four independent variables, they were all included in the multivariable model. P values of <0.05 were considered statistically significant.

### Ethics

Ethical approval was provided by the Rwandan National Ethics Committee and the research ethics committee of the Academic Medical Center, Amsterdam. All patients are aware that as part of clinical care their demographic and clinical data are registered in paper-based clinic registers. As only such routinely collected data were abstracted from clinic registers, and no names were entered into the electronic study database the ethics committees did not require that written informed consent was sought from patients.

## Results

Of the 525 patients diagnosed with HIV in the eight clinics between 1^st^ March and 31^st^ May 2009, 43 were excluded from the analysis due to non-matching link codes between the registers or missing patient names, leaving 482 HIV patients for analysis (Figure 2).

### Enrolment in care and treatment

Table 1 shows the number of newly diagnosed HIV patients by department, age group, sex, and study site. The majority (293; 61%) of HIV patients were diagnosed in VCT departments. Seventy percent of the study patients (339) were female. The median age of patients was 29 years (interquartile range [IQR] 24–37). Almost one third of all patients were diagnosed at Muhoza clinic, a large urban clinic in north-west Rwanda.

Among the 482 patients, 201 (42%) enrolled in care and treatment within 90 days of HIV diagnosis (see Table 2). For those enrolled within 90 days of HIV testing, the median time (IQR) between HIV testing and enrolment into care and treatment was 6 days (2–14). After seven days 115 patients (24%) had enrolled, by 30 days 180 (37%) and by 60 days 193 (40%) (see Figure 3.a). Twenty-one patients (4%) enrolled after 30 days and only eight (2%) after 60 days. There was a significant variation in the proportion of patients enrolled in care and treatment between study sites (*p*<0.001). Patients at all other sites other than Muhoza (the largest clinic) were more likely to be enrolled within 90 days (50% vs. 24%, *p*<0.001). Similar proportions of males and females enrolled in HIV care and treatment (43% and 41% respectively; *p* = 0.6). Age was not significantly associated with enrolment (*p* = 0.25). Differences in enrolment between departments where the HIV diagnosis was made were not statistically significant (*p* = 0.14).

When we defined enrolment not as being registered at the ARV clinic but as a CD4 count having been done, the proportion enrolled was 39% (188/482) and median time to enrolment among those who enrolled was 6 days (2–13 days).

### CD4 count and WHO stage

Of the 201 HIV patients that enrolled in care and treatment, WHO staging and CD4 counts were done for 89% (179/201) and 94% (188/201) respectively; both WHO stage and CD4 count were available in 173 patients (86%). Three of these 173 patients were less than 15 years of age and were excluded from further analysis. The medium time between HIV diagnosis and CD4 count was 6 days (IQR 2–13).

The median CD4 count among 185 adult patients was 387 cells/mm^3^ (IQR: 242 to 533 cells/mm^3^). Forty-three percent has a CD4 count below 350 cells/mm^3^; 40 patients (22%) had a CD4 count below 200 cells/mm^3^. The median CD4 count of males was lower than that of females (330 vs. 412 cells/mm^3^; *p* = 0.01; see Table 2). Patients diagnosed at OPDs and TB units had lower median CD4 counts (respectively 287 and 188 cells/mm^3^) than those enrolled at VCT or ANC (*p* = 0.0002).

In univariable analysis, low CD4 count (defined by a CD4 count <350 cells/mm^3^) was significantly associated with department, age, and sex (*p* = 0.006, *p* = 0.008, and *p* = 0.03 respectively), but not with study site (see Table 3). Multivariable logistic regression analysis revealed only age as an independent factor: older age was associated with lower CD4 counts (*p* = 0.02).

Among the 198 enrolled adult patients, 107 were in WHO stage 1, 41 in stage 2, 28 in stage 3 and none were in stage 4 (for 22 patients no WHO stage was recorded). WHO stage 3 or 4 was associated with study site, department and age (all *p*<0.005), but not with sex (*p* = 0.17). In multivariable logistic regression analysis department of HIV diagnosis was significantly associated with an advanced WHO stage (those diagnosed at ANC being less likely and those diagnosed at the TB department being more likely to have WHO stage 3/4); older age was associated with advanced WHO stage. As there were no patients in WHO stage 4, the analysis of determinants for ART eligibility was basically identical to the one analysing determinants of low CD4 counts (see above).

### Start of ART

Of the 81 eligible patients 45 (56%) started ART within 90 days of HIV diagnosis. For those who started ART within 90 days, the median time (IQR) between date of HIV diagnosis and start of ART was 30 days (18–43). After seven days none of the patients had started ART, by 30 days 24 (30%) and by 60 days 41 (51%) (see Figure 3.b). Twenty-one of eligible patients (25%) started ART more than 30 days after HIV diagnosis, but only 4 (5%) started ART after 60 days For those who started ART within 90 days, the median time between date of CD4 count and start of ART was 22 days (IQR 13–37). A statistically significant association was observed between the study site and the start of ART (*p*<0.001; see Table 2). At three of the eight study sites all ART eligible patients started treatment within 90 days of HIV diagnosis; at one site less than 20%. A higher proportion of eligible females than males started ART (63% vs. 46%), but this was not significant (*p* = 0.13). Neither department nor age were associated with start of ART (*p* = 0.64 and *p* = 0.52 respectively). Numbers were too small to perform multivariable analysis.

### Children

There were 10 children among the 482 patients: four boys and six girls. Their median age was 3 years (range 1–9 years old). The children were identified in six sites; two of them were tested in OPD and eight at the VCT department. Only three of the ten children enrolled at care at ARV clinics, were staged and had CD4 counts measured. One of them had a low CD4 count and was in stage 2; the other two had normal CD4 counts and were in stage 1. The child with low CD4 count started ARV, 37 days after HIV diagnosis.

## Discussion

### Main findings

Following HIV diagnosis, registration at ART clinics is crucial for assessment of treatment eligibility, counselling, and start of ART. This study shows that a low proportion of HIV patients (42%) attended ART clinics for ART eligibility assessment and start of care and treatment within 90 days of HIV diagnosis. About half of enrolled patients were eligible for ART, and just over half those actually started ART within 90 days of diagnosis.

### Enrolment

Other studies from sub-Saharan Africa (SSA) among people diagnosed with HIV at VCT centres or out-patient departments [4], [8], [13]–[15] reported higher enrolment proportions, varying from 55% in Durban (South Africa) [4] to 68% in South West Uganda [8]. One study from South Africa reported a much higher proportion, 85%, but in that setting CD4 counting was done at the same visit as the HIV test [16]. A study from Lusaka, Zambia, reported a much lower proportion, 38% [6].

Studies have used different cut-offs to calculate enrolment (varying between 8 weeks and 6 months), but these differences do not explain the variations in enrolment, as the median time to enrolment tends to be counted in days rather than weeks, and few are enrolled more than 4 weeks after the HIV diagnosis [6], [7], [13] We defined enrolment into care and treatment as a first visit and registration at the ART clinic. If we had used the definition of a recent meta-analysis (date of CD4 count) [15] the enrolment would have been 39% rather than 42%; this compares to the median of 59% found in the systematic meta-analysis by Rosen & Fox [15].

Most other studies, like ours, were conducted in public health programs. The low overall enrollment in our study was strongly influenced by one large clinic with only 24% enrolment. The enrolment in all other clinic combined was 50%. The clinic with the low enrolment rate was the only site that had to refer patients off-site to an ARV clinic. Neither age nor sex of patients was associated with enrolment, but health facility was. This suggests that health services should be improved to enable a more successful transfer of patients from diagnosing clinic to ART clinic. It also suggests that presence of an ARV clinic at the HIV testing site may be conducive for high enrolment. This appears a relatively straightforward and achievable approach to increase enrolment and uptake of ART.

### Eligibility

The median CD4 count of the HIV patients who enrolled into care was quite high (387 cells/mm^3^) compared to the average CD4 count in many other HIV programs in sub-Saharan Africa [17] and elsewhere. At the time of this study (2009) Rwanda already used the 350 cells/mm^3^ cut-off for treatment eligibility. Thus, in spite of the generally high CD4 count in the study population, less than half of the enrolled patients in our study were eligible for immediate ART. Few patients were in very advanced stage of infection (none in WHO stage 4 and only 9% with CD4 count below 100 cells/mm^3^). Provider-initiated testing could potentially lead to early HIV diagnosis and a timely start of ART for even more people.

### Start of antiretroviral treatment

Fifty-six percent of eligible patients had started ART within 90 days of HIV diagnosis. This proportion is too low and indicates delays. Other studies from SSA [5], [13], [14], [18] reported that between 67% [14] and 86% [18] of eligible patients started ART within 3 to 6 months. If we restrict our analysis to those who are eligible based on the criterion used in most other studies (<200 cells/mm^3^), [5], [13], [19] only 22% of patients would have been eligible; 68% of these started ART <90 days after diagnosis, which is more in line with other studies done in sub-Saharan Africa.

Like many other studies in SSA [19]–[22] we found that a much higher proportion of men than women (61% vs. 41%; *p* = 0.016) was eligible for immediate start of ART. This indicates that men are diagnosed later in their HIV disease course than women. This may be a reason for the higher mortality among HIV infected men on ART, observed in other studies [23]–[26]. Older patients were at a more advanced disease stage than young people at the time of diagnosis, both in terms of clinical condition (WHO stage) and in terms of CD4 count. This finding was independent of sex, so is not just caused by the large number of, predominantly younger, pregnant and delivering women. More testing of older adults (aged above 35 years) should be encouraged and PIT has the potential to address this issue.

### Universal access and linkage to care and treatment in Rwanda

A recent report of WHO/UNAIDS indicated that worldwide only three countries with a generalized HIV epidemic have achieved universal access of ART (defined as providing antiretroviral therapy to at least 80% of the people eligible for treatment), Rwanda being one of those three countries [2]. Our finding of low linkage to care and treatment after HIV diagnosis may appear paradoxical in view of this remarkably high ART coverage. There may be several explanations for this. In Rwanda HIV testing rates among the population are high and it is thought that the majority of HIV infected people know their status. This is the case for those eligible for treatment as well as those not yet eligible. Nevertheless, a significant proportion of people have never been tested. If the people diagnosed with HIV infection in our study were not previously aware of their status, it is to be expected that a fair proportion of them will be eligible for immediate ART. Qualitative research by our group (unpublished; work in progress) found that several patients being tested for HIV at health facilities had already had a positive HIV test before. Some sought another HIV test because they did not believe the initial positive result; others hoped they would have cleared the infection spontaneously; and some had initially been tested at another clinic than their own neighbourhood clinic. Due to this “repeat positive testing”, the observed enrolment may be an underestimate. Our results suggest that even in a program with very high coverage of ART, improvements can still be made.

### Limitations

Our study was subject to certain limitations. Some errors in patient identifiers (names, age, sex, department of initial consultation) between registers posed a problem of identification of some study patients. This resulted in the exclusion of 8% of patients. Field staff may have overlooked names in the ART registers of people that were actually on their list of HIV infected patients, due to unclear writing, variations in spelling of names, or usage of different names. In some cases patients did start ART but dates of start were missing; this contributed to a lower estimated proportion of patients that started ART. The selection of health facilities was not random, so the findings may not be generalizable to Rwanda as a whole. It is unlikely that patients attended other ART clinics than the ones they were referred to, as no functional referral system between clinics was in place at the time of the study; patients presenting at another clinic than the one they were originally tested at, would normally be tested again for HIV at that clinic before they would be registered, assessed for eligibility or started on ART. Possibly the requirement of disclosure of HIV status to family members prior to HAART initiation may have played a role in the low enrolment rate, but as this analysis used routine clinic data we were unable to assess this.

The main strength of our study is that we used routine data from primary care clinics and hospitals, reflecting routine health care practice, rather than using data from research clinics where quality of referral and assessment may be better.

### Conclusion

Less than 50% of people were enrolled at an HIV care clinic within 90 days of their HIV diagnosis. We found no association between age or sex of the patient and enrolment, but enrolment varied substantially between health facilities. Thus, improving linkage between clinics of testing and ART clinics could increase ART coverage. Just over half of eligible patients started ART within 90 days of diagnosis. Targeted efforts to improve pre-ART patient retention are needed.
